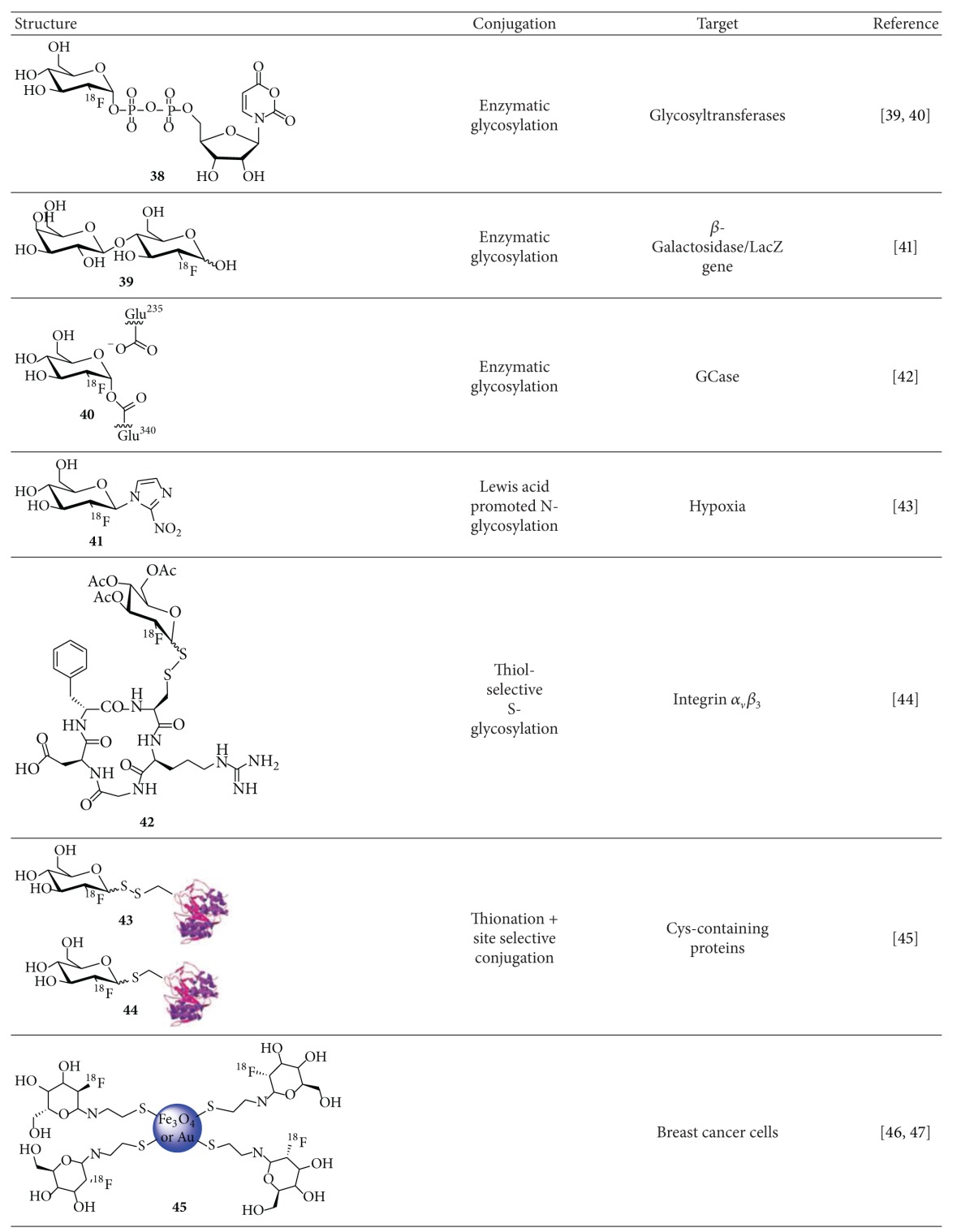# Sweetening Pharmaceutical Radiochemistry by ^18^F-Fluoroglycosylation: A Short Review

**DOI:** 10.1155/2014/214748

**Published:** 2014-06-01

**Authors:** Simone Maschauer, Olaf Prante

**Affiliations:** Molecular Imaging and Radiochemistry, Department of Nuclear Medicine, Friedrich Alexander University, Schwabachanlage 6, 91054 Erlangen, Germany

## Abstract

At the time when the highly efficient [^18^F]FDG synthesis was discovered by the use of the effective precursor 1,3,4,6-tetra-*O*-acetyl-2-*O*-trifluoromethanesulfonyl-**β**-D-mannopyranose (mannose triflate) for nucleophilic ^18^F-substitution, the field of PET in nuclear medicine experienced a long-term boom. Thirty years later, various strategies for chemoselective ^18^F-labeling of biomolecules have been developed, trying to keep up with the emerging field of radiopharmaceutical sciences. Among the new radiochemical strategies, chemoselective ^18^F-fluoroglycosylation methods aim at the sweetening of pharmaceutical radiochemistry by providing a powerful and highly valuable tool for the design of ^18^F-glycoconjugates with suitable *in vivo* properties for PET imaging studies. This paper provides a short review (reflecting the literature not older than 8 years) on the different ^18^F-fluoroglycosylation reactions that have been applied to the development of various ^18^F-glycoconjugate tracers, including not only peptides, but also nonpeptidic tracers and high-molecular-weight proteins.

## 1. Introduction

In 1984, thirty years ago, the synthesis of 1,3,4,6-tetra-*O*-acetyl-2-*O*-trifluoromethanesulfonyl-*β*-D-mannopyranose (mannose triflate) was published by Hamacher in* Carbohydrate Research* [1]. Meanwhile, the mannosyl precursor is commercially available and its daily routine application for the highly reliable and efficient radiosynthesis of 2-deoxy-2-[^18^F]fluoroglucose ([^18^F]FDG, [2]) has been the major driving force in the emerging field of positron emission tomography (PET) within nuclear medicine. [^18^F]FDG represents by far the most frequently used radiopharmaceutical worldwide for PET imaging studies in oncology and neurology [3, 4].

Among the positron emitters, F-18 represents a superior PET radionuclide with outstanding physical characteristics (*E*_max⁡_(*β* ^+^) = 635 keV, *t*_1/2_ = 109.7 min) allowing for multistep radiochemical syntheses. In recent years, various strategies for chemoselective ^18^F-labeling reactions have been successfully developed, facilitating the accessibility of new PET radiopharmaceuticals [5–7]. The variety of new ^18^F-labeling strategies is the focus of review articles as part of this special issue, provided by Bernard-Gauthier et al. [8], Kettenbach et al. [9], and Ermert [10].

In some cases it is desirable not only to use chemoselective and mild labeling methods, but also to have the opportunity to influence the biodistribution and tracer uptake characteristics simultaneously. Noteworthy, the glycosylation of biomolecules, such as peptides or proteins, has been frequently shown to improve the* in vivo* kinetics and stability in blood and to accelerate the clearance of such glycoconjugates* in vivo* [11–13]. Moreover, it has been shown by numerous examples that glycosylation of peptides with subsequent radiolabeling opens the way to radiotracers with improved* in vivo* properties [12–16]. Not surprisingly, Sharpless' concept of “click chemistry” introduced over 10 years ago [17] has been quickly adapted to carbohydrate chemistry in the field of glycoscience, facilitating the synthesis of new glycoconjugates derived from proteins, lipids and nucleic acids, new glycomaterials, such as glycosurfaces, glycodendrimers, and glycopolymers, and a wide variety of glycoconjugates in medicinal chemistry as putative chemotherapeuticals [18].

Therefore, our group envisaged the appropriate idea to develop a click chemistry-based ^18^F-fluoroglycosylation method to provide a general approach to the radiosynthesis of ^18^F-labeled glycoconjugates as effective imaging agents for PET [19, 20].

In 2009, we reported the synthesis of a series of “clickable” mannosides and showed their potential as ^18^F-labeling precursors [20], which has been the starting point of a number of publications by others reporting on various ^18^F-glycoconjugates as new PET tracers.

This paper provides a short review (reflecting the literature not older than 8 years) on the different ^18^F-fluoroglycosylation reactions that have been applied to the development of various ^18^F-glycoconjugate tracers for PET, including not only peptides but also nonpeptidic tracers and high-molecular-weight proteins. An overview of the radiotracers obtained by ^18^F-fluoroglycosylation is given in Tables 1, 2 and 3.

## 2. ^**18**^F-Fluoroglycosylation via Copper-Catalyzed Azide-Alkyne Cycloaddition (CuAAC)

In 2001, the Sharpless group introduced the term “click chemistry” to define the most efficient chemical reactions [17]. Click chemistry-based reactions are easy to perform, high-yielding, highly chemoselective, and orthogonal reactions that proceed without the formation of by-products under multiple reaction conditions. The copper-catalyzed azide-alkyne cycloaddition (CuAAC), introduced in 2002 [48, 49], is one of the most widely used click chemistry reactions, due to its high yield and easy accessibility of the azide and terminal alkyne reactants. Its successful adaption to ^18^F-radiosynthetic methods in order to take advantage of its high selectivity, reliability, and speed under aqueous mild Cu^I^-promoted reaction conditions has now been amply documented [50, 51].

**Scheme 1:**
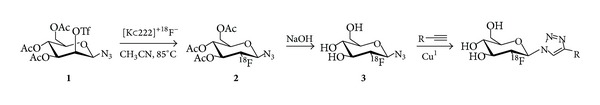
General reaction scheme for [^18^F]fluoroglycosylation via CuAAC using 2-deoxy-2-[^18^F]fluoroglucopyranosyl azide** 3**, starting from the *β*-mannosyl azide** 1**.

The synthesis of the ^18^F-fluoroglycosylating agent 3,4,6-tri-*O*-acetyl-2-deoxy-2-[^18^F]fluoroglucopyranosyl azide (**3**) was achieved by the ^18^F-labeling of the precursor 3,4,6-tri-*O*-acetyl-2-*O*-trifluoromethanesulfonyl-*β*-D-mannopyranosyl azide (**1**) in high radiochemical yields (RCY) as described by Maschauer and Prante (Scheme 1) [20]. Very similar to the well-known [^18^F]FDG synthesis, the yield of this radiochemical reaction relies mainly on the chemical purity of the labeling precursor. In the case of mannosyl azide** 1**, the synthesis was achieved by comprehensive carbohydrate chemistry via the pentafluoropropionyl protected *β*-mannosyl bromide and the purification of** 1** was achieved by recrystallization in ethanol. The radiolabeling reaction according to Scheme 1 could be performed under standard reaction condition (Kryptofix 222, K_2_CO_3_) or with a slight modification using a mixture of K_2_CO_3_/KH_2_PO_4_ so that the solution is less basic and therefore the degradation of the mannosyl precursor** 1** during the labeling reaction is significantly reduced, resulting in a more accurate HPLC separation of** 2** without the interference of by-products. The CuAAC could also be performed with high RCY by omitting the HPLC separation. However, the presence of precursor azide** 1** in the CuAAC reaction required the high alkyne concentration of 33 mM, making it inefficient for most rare peptides.

The applicability of the prosthetic group** 3** for CuAAC was first verified with alkyne-bearing amino acids [20]. In ongoing studies the methodology was transferred to the radiosynthesis of an ^18^F-fluoroglycosylated RGD peptide (**4**) and a neurotensin peptoid (**5**) [19]. The optimized CuAAC was carried out in PBS/ethanol (10 : 1, v/v) at 60°C with only 0.2 mM alkyne-peptide in the presence of CuSO_4_ (4 mM) and sodium ascorbate (12 mM). After 15–20 min the reaction was complete and the ^18^F-glycopeptides were isolated by HPLC in overall (non-decay-corrected) yields of 17–20% in a total synthesis time of only 70–75 min (starting form [^18^F]fluoride) with specific activities of 55–210 GBq/*μ*mol.

Furthermore, this ^18^F-fluoroglycosylation approach was adopted in the radiosynthesis of ^18^F-glycoproteins [21]. The first reaction step (^18^F-labeling of the precursor** 1**, Scheme 1) was performed under standard reaction conditions and yielded 3,4,6-tri-*O*-acetyl-2-deoxy-2-[^18^F]fluoroglucopyranosyl azide** 2** after HPLC separation in surprisingly low yields of only 1.3–4.7% after 80–100 min. After deacetylation the solution was neutralized by passing it through a cation exchange resin and the CuAAC was performed with an alkyne-bearing protein (6 *μ*M) in the presence of copper(I) bromide and a tris-triazolyl amine ligand (triethyl 2,2′, 2′′-[nitrilotris(methylene-1*H*-1,2,3-triazole-4,1-diyl)]triacetate, TTMA) in sodium phosphate buffer (pH 8.2)/acetonitrile (6 : 1, v/v). After 45 minutes at room temperature the RCY of** 6** was 4.1%. The observed low RCY is clearly due to the extremely low amount of the alkyne-bearing protein that was not available in higher amounts since it was a mutated protein that was produced in bacteria using site-directed mutagenesis of the protein gene.

In an approach toward the synthesis of selective PET ligands for the neurotensin receptor subtype-2 (NTS2), Held et al. synthesized a series of NT(8-13) peptide-peptoid hybrids with* N*-homo-Tyr instead of Tyr^11^ and variations of the original Arg^8^-Arg^9^ leading to peptides with very high NTS2 affinity and selectivity over NTS1 [22]. The sequence* N*lys-Lys-Pro-*N*-homo-Tyr-Ile-Leu-OH was further derivatized at the* N*-terminus with propargylglycine making it suitable for CuAAC with 2-deoxy-2-fluoroglucopyranosyl azide and 6-deoxy-6-fluoroglucopyranosyl azide. Surprisingly, both analogues** 7** and** 8** showed a dramatic loss of NTS2 affinity compared to the nonglycosylated compound (110–290 nM versus 4 nM), rendering this approach for the development of NTS2-selective PET ligands unfavorable.

The ^18^F-glycosylation via CuAAC was also transferred to the radiosyntheses of a couple of nonpeptidic compounds [23–29]. For the radiosynthesis of an ^18^F-fluoroglycosylated folate derivative by Fischer et al., the intermediate 3,4,6-tri-*O*-acetyl-2-deoxy-2-[^18^F]fluoroglucopyranosyl azide** 2** was separated by solid phase extraction, excluding the HPLC purification [23]. After hydrolysis, the CuAAC with folate alkyne was performed in aqueous ethanol (38%) in the presence of Cu(OAc)_2_ (1.2 mM) and sodium ascorbate (2.4 mM). After 15 min at 50°C the conversion was complete and the ^18^F-labeled product was obtained after HPLC isolation in overall RCY of up to 25%, with a specific activity of 90 ± 38 GBq/*μ*mol. Unfortunately, the authors did not provide any information on the amount of folate alkyne needed for the CuAAC reaction. The stability of [^18^F]FDG-folate** 9** was analyzed in human serum and murine liver microsomes and did not reveal any defluorination or radioactive degradation products within 120 and 60 min, respectively, at 37°C. Analyses of plasma, urine, and liver tissue at 30 min postinjection (p.i.) in mice confirmed high tracer stability* in vivo*. In addition, biodistribution and small-animal PET studies were performed in nude mice bearing folate receptor- (FR-) expressing KB-tumors. High specific uptake and retention were found in the KB tumor and in all organs with known FR expression (i.e., kidneys and the salivary glands) from 30 to 90 min p.i. The blood clearance was fast, resulting in tumor-to-blood ratios of 36 ± 15 at 90 min p.i. Although the log⁡⁡*D*_7.4_ value of −4.2 indicated high hydrophilicity of the compound, a high nonspecific accumulation in liver and gall bladder was observed, possibly due to a carrier-mediated uptake of the folic acid derivative** 9** into the hepatocytes.

Recently, an improved ^18^F-fluoroglycosylated folate conjugate with an albumin binding entity has been reported by the same group [24]. This study aimed at the enhancement of the blood circulation time of the tracer by an albumin-binding moiety and hence improvement of the tumor-to-kidney ratio of the radiotracer uptake. The conjugate was radiolabelled via CuAAC using 2-deoxy-2-[^18^F]fluoroglucopyranosyl azide** 3** and the alkyne-functionalized folate precursor (2.2 mM) in the presence of Cu(OAc)_2_ (1 mM) and sodium ascorbate (3 mM) in water/DMF (60 : 40) at 50°C for 15 min in a RCY of 15%. The HPLC separated product** 10** was obtained in an overall RCY of only 1-2% after a total synthesis time of 3 h in specific activities of 20 to 50 GBq/*μ*mol. Biodistribution and PET studies on KB tumor-bearing nude mice** 10** revealed a slow blood clearance with uptake values of 2.2% ID/g at 4 h p.i., substantially high tumor uptake values of 11–15% ID/g at 1–4 h p.i., and improved tumor-to-kidney ratios of about 1. Similar to the previously published folate** 9** this albumin-binding tracer (**10**) also showed very high nonspecific uptake in the gall bladder.

A series of inhibitors for the matrix metalloproteinases (MMPs) MMP-2, MMP-8 MMP-9, and MMP-13 as tools for the visualization of activated MMPs with PET were developed by Hugenberg et al. [25]. Therefore, the hydroxamate-based lead structures CGS 27023A and CGS 25966 were triazole-substituted resulting in several mini-PEG-derivatized and glycosylated ligands. From all compounds the inhibition potencies were determined and log⁡⁡*D*_7.4_ values were calculated. The fluoroglycosylated compound displayed a log⁡⁡*D*_7.4_ of 0.58 and subnanomolar inhibition potencies (0.2–0.6 nM) for the various MMPs, rendering the corresponding ^18^F-glycoconjugate a potential PET tracer candidate. However, the fluoroethyl-1,2,3-triazole derivative had a calculated log⁡⁡*D*_7.4_ of 1.53 and revealed outstanding inhibition potencies of 0.006–0.13 nM (for MMP-2, -8, -9, and -13), so this compound was chosen for radiolabeling and further studies.

An example where fluoroglycosylation leads to a complete loss of affinity was reported by Banerjee et al. [26]. In their search for subtype selective dopamine D4 receptor radioligands, a series of* N*-aryl piperazinyl methyl triazoles bearing fluorine-substituted appendages was synthesized and the target compounds were investigated for dopamine and serotonin receptor binding. With the aim of biasing the hydrophilicity and optimizing the D4 receptor affinity and selectivity, a concise series of triazoles containing fluoroalkyl, fluoroalkoxy, fluoroalkoxyphenyl, and deoxyfluoroglucosyl substituents was studied. The glycosylated compounds** 12** and** 13** had low calculated log⁡*P* values of about 0, but affinities for the D4 receptor of 500 nM and 340 nM, respectively, which are 100 and 66 times lower when compared to the fluoropropoxyphenyl compound (5.1 nM) which had the highest affinity for D4 in this series.

The ^18^F-fluoroglycosylation by CuAAC was also used for the radiosynthesis of an ET_A_ receptor (ET_A_R) ligand [27]. Therefore the fluoroglycosyl moiety was introduced into the lead compound PD156707 as a hydrophilic building block. For the radiosynthesis of the glycoconjugate (**14**), the appropriate alkyne (0.6 mM) was allowed to react with ^18^F-glucosyl azide** 3** in saline/ethanol (3 : 2, v/v) in the presence of sodium ascorbate (12 mM) and CuSO_4_ (4 mM), providing** 14 **in high non-decay-corrected yields (20–25%, 70 min) and a specific activity of 41–138 GBq/*μ*mol. The triazolyl conjugated fluoroglucosyl derivative had high selectivity for ET_A_R (4.5 nM) over ET_B_R (1.2 *μ*M). The high metabolic stability of the glycoconjugate was demonstrated by HPLC analysis of extracts from mouse blood and gall bladder collected at 60 min p.i. Biodistribution studies on K1 tumor-bearing nude mice revealed** 14** to have fast blood clearance, low uptake in the kidneys and liver, but a very high uptake in the bile and intestines. This indicates that despite glycosylation the tracer is predominantly excreted via hepatobiliary clearance, a finding in accordance with previously studied ^18^F-derivatives of PD 156707.

The radiosynthesis of a diarylpyrazole glycoconjugate, derived from the potent NTS1 antagonist SR142948A, was also successfully performed by the click ^18^F-fluoroglycosylation using the CuAAC reaction [28]. This nonpeptidic NTS1 ligand was achieved by allowing** 3** to react with the alkyne-bearing diarylpyrazole precursor (0.3 mM) in saline/tetrahydrofuran (3 : 4, v/v) for 10 min at 60°C. The ^18^F-fluoroglycosylation proceeded in a total synthesis time of 70 min, and the ^18^F-glycoconjugate (**15**) was obtained in a non-decay-corrected yield of 20 ± 3% and a specific activity of 35–74 GBq/*μ*mol. The log⁡⁡*D*_7.4_ was determined to be −0.24. The glycoconjugate** 15 **displayed excellent affinity toward NTS1 (*K*_i_ = 1 nM) and substantial stability* in vivo*. Biodistribution and PET studies in nude mice bearing NTS1-expressing HT29 tumors demonstrated excellent tumor retention with an uptake of 0.84% ID/g at 10 min p.i. and 0.74% ID/g at 60 min p.i. and fast clearance from blood and all other organs resulting in a tumor-to-blood ratio rapidly increasing from 0.3 to 4.4 from 10 to 60 min p.i.

In a study reported by Pisaneschi et al. 2-deoxy-2-[^18^F]fluoroglucopyranosyl azide** 3** was used for the radiosynthesis of a new ^18^F-fluoroglycosylated cyanoquinoline for PET imaging of epidermal growth factor receptor (EGFR) [29]. In this study** 3** was not isolated by HPLC, but only separated via SPE. Subsequently, the CuAAC was performed with the alkyne precursor (3 mM) in a mixture of PBS/acetonitrile (96 : 4, v/v) in the presence of CuSO_4_ (10 mM), sodium ascorbate (66 mM), and bathophenanthroline disulfonic acid disodium salt (BPDS) (6.7 mM), as an additive to stabilize Cu(I) oxidation state, at room temperature for 5 min in the RCY of about 50%. The final ^18^F-labeled glycoconjugate** 16** was isolated by semipreparative HPLC and obtained in 9% non-decay-corrected yield (starting from [^18^F]fluoride) after a total synthesis time of 90 min with a specific activity of 7.3 GBq/*μ*mol.** 16** was tested* in vitro* in a cellular uptake experiment using A431 cells, harbouring high EGFR expression, in comparison with low EGFR-expressing MCF7 cells, demonstrating selective uptake in EGFR-positive cells.

**Scheme 2:**
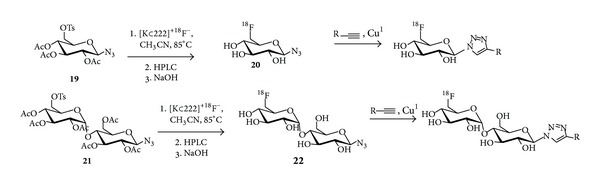
General reaction scheme for ^18^F-fluoroglycosylation via CuAAC using 6-deoxy-6-[^18^F]fluoroglucopyranosyl azide** 20** and 6′-deoxy-6′-[^18^F]fluoromaltosyl azide** 22**; a recent example for the alkyne is cyclic peptide c(RGDfPra), as reported by Maschauer et al. [30].

Very recently, the reliability and robustness of the above described ^18^F-fluoroglycosylation strategy prompted us to extend the series of ^18^F-fluoroglycosyl azides by introducing 6-deoxy-6-[^18^F]fluoroglucopyranosyl azide and 6′-deoxy-6′-[^18^F]fluoromaltosyl azide (Scheme 2) [30]. Both compounds were synthesized from their corresponding peracetylated 6-tosylate precursors** 19** and** 21** in high RCY of 84% and 61%, respectively. The acetylated intermediates were isolated by HPLC and subsequently hydrolyzed with NaOH (60 mM) to give the “clickable” glycosyl azides** 20** and** 22**. The CuAAC was performed with alkyne-bearing RGD-peptide c(RGDfPra) (0.3 mM) in saline/ethanol in the presence of CuSO_4_ (4 mM), sodium ascorbate (10 mM) at 60°C for 15–20 min. The RCY of this step was about 80% for both [^18^F]fluoroglycosyl derivatives** 17** and** 18** with specific activities of 50–200 GBq/*μ*mol; the overall yield was 16–24% (non-decay-corrected, starting from [^18^F]fluoride) within a total synthesis time of 70–75 min. Both ^18^F-glycopeptides** 17** and** 18** (abbreviated by [^18^F]6Glc-RGD and [^18^F]Mlt-RGD, [30]) were studied* in vivo* using U87MG tumor-bearing nude mice and compared to the previously published 2-deoxy-2-[^18^F]fluoroglucopyranosyl RGD derivative** 4** ([^18^F]2Glc-RGD, [19]). It was observed that [^18^F]6Glc-RGD (**17**) and [^18^F]Mlt-RGD (**18**) showed significantly decreased liver and kidney uptake relative to [^18^F]2Glc-RGD (**4**). More importantly, [^18^F]Mlt-RGD (**18**) revealed substantial tumor uptake and high retention in the U87MG tumors comparable to that of [^18^F]galacto-RGD [14, 52], resulting in tumor-to-kidney ratios comparable with some dimeric RGD peptides [53, 54]. Its favorable biodistribution together with excellent clearance properties* in vivo* makes [^18^F]Mlt-RGD (**18**) a viable alternative PET tracer for imaging integrin expression.

## 3. ^**18**^F-Fluoroglycosylation via Oxime Formation

The principle advantages of oxime formation by click reaction between an aminooxy- and a carbonyl functionality for ^18^F-fluoroglycosylation are its high chemoselectivity, the use of unprotected aminooxy precursors, and the fact that coupling with the carbonyl component can be performed in aqueous media (pH 4–7). The formed oxime occurs in *E*- and *Z*-form in solution, both being stable under physiological conditions. The resulting *E*/*Z* isomeric ratio of an oxime depends on the size of substitutes at the C = N double bond. However, as the two isomers equilibrate very quickly in solution, the *E*- and *Z*-forms are usually not isolated from each other, but collectively considered as one compound.

**Scheme 3:**
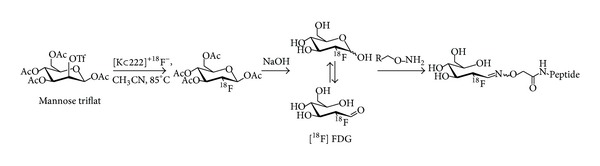
General reaction scheme for ^18^F-fluoroglycosylation via oxime formation using [^18^F]FDG.

**Scheme 4:**
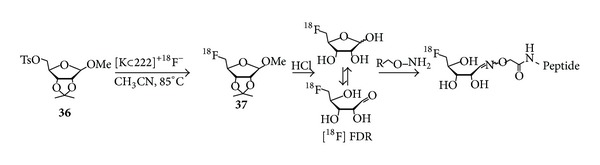
General reaction scheme for ^18^F-fluoroglycosylation via oxime formation using [^18^F]FDR.

[^18^F]FDG can be used for a fluoroglycosylation reaction by oxime formation, because in aqueous solutions it undergoes mutarotation; that is, [^18^F]FDG isomerizes between the *α*- and *β*-anomer through the intermediate acyclic aldehyde (Scheme 3). The mutarotation equilibrium is sometimes described to be favored at high temperatures (80–120°C) and to be more efficient at acidic pH (1.5–2.5) [32, 33, 35]. This is a drawback when using large peptides which can undergo degradation under such high temperatures and acidic conditions. The use of 5-[^18^F]fluoro-5-deoxyribose ([^18^F]FDR) (Scheme 4) could compensate for these limitations because the location of the fluorine at C-5 of the 5-membered ring might facilitate the formation of the acyclic form of [^18^F]FDR making it possible to perform the oxime formation at room temperature at pH 6.0 with high yields [37].

An approach to use [^18^F]FDG not for direct radiolabeling but for the preparation of a maleimidehexyloxime prosthetic group ([^18^F]FDG-MHO) for the chemoselective ^18^F-labeling of thiol-containing peptides and proteins was reported in 2008 by Wuest et al. [31]. [^18^F]FDG-MHO was prepared by conjugation of [^18^F]FDG with aminooxymaleimide hydrochloride (40 mM) in saline/ethanol (1 : 5) at 100°C for 15 min. After HPLC isolation, [^18^F]FDG-MHO was obtained in 42% RCY (based upon [^18^F]FDG) in a synthesis time of 45 min. The conjugation with the 36 kDa, single thiol group-containing protein annexin-V was performed in tris-buffer (pH 7.4)/ethanol (1 : 5, v/v) at room temperature for 30 min. Using only low amount of the protein (22 *μ*M), [^18^F]FDG-MHO-anxA5 (**23**) was obtained after size-exclusion chromatography in RCY of 43–58% (based upon [^18^F]FDG-MHO) within 60 min and in specific activities of 2–4 GBq/*μ*mol.

The first studies using [^18^F]FDG as prosthetic group for the direct labeling of aminooxy-functionalized peptides were published in 2009 from two different groups [32, 33]. The group of Gambhir reported the synthesis of an ^18^F-labeled linear RGD peptide ([^18^F]FDG-RGD,** 24**) and a cyclic RGD peptide ([^18^F]FDG-cyclo(RGDyK)) as the first examples for the use of [^18^F]FDG in the oxime formation with aminooxy-peptides [32]. They prepared [^18^F]FDG-RGD (**24**) and [^18^F]FDG-cyclo(RGDyK) within 60–70 min in 27.5% and 41% overall RCY (based on [^18^F]FDG), respectively. [^18^F]FDG was allowed to react with aminooxy-functionalised RGD (48 mM) or aminooxy-functionalised c(RGDyK) (24 mM) in TFA (0.4%) in saline or TFA (0.4%)/ethanol (16%) in saline at 100°C for 30 or 45 min, respectively. They found that under these experimental conditions maximum RCY was obtained at pH values of 1.5–2.5. When the reaction was performed at pH 4 in ammonium acetate buffer, no significant products were produced. Both ^18^F-labeled glycopeptides were isolated by radio-HPLC and used for small-animal PET studies with U87MG-xenografted nude mice. At 120 min p.i. the uptake of** 24** in the U87MG tumor was quite low (0.3% ID/g), whereas the uptake in heart was very high (3.7% ID/g). [^18^F]FDG-cyclo(RGDyK) showed increasing uptake in the kidneys (4.7–12% ID/g) and in the tumor (0.6–1.5% ID/g) over time (30–120 min) with tumor-to-blood ratios of only 3 after 120 min. Unfortunately, the authors did not provide any information on the specific activity of the ^18^F-labeled glycopeptides.

Simultaneously, the use of [^18^F]FDG in oxime formation with peptide derivatives was published by Hultsch et al. [33]. They found that the use of clinical grade [^18^F]FDG containing about 200 *μ*g/mL glucose did not allow the radiosynthesis of [^18^F]FDG-RGD (**25**) in sufficient yield. The use of a small volume of [^18^F]FDG (37 MBq) corresponding to a molar ratio of peptide to D-glucose in the reaction mixture of approximately 40 and a concentration of the aminooxy-functionalized peptide of 35–50 mM leads to a high RCY of 73% for the oxime formation of** 25**; however, this procedure is restricted to maximal yields of only 37 MBq. When using a 10 times larger volume of [^18^F]FDG (370 MBq) corresponding to a peptide-to-glucose ratio of 4 and a peptide concentration of 4-5 mM, almost no RCY was obtained. Instead, the nearly exclusive formation of the nonradioactive D-glucose-RGD oxime conjugate was observed. Therefore, n.c.a. [^18^F]FDG was produced by separation of the clinical grade [^18^F]FDG from excess glucose by radio-HPLC. Following this strategy, the conjugation of n.c.a. [^18^F]FDG with Boc-protected aminooxyacetyl-conjugated c(RGDfK) (5 mM) was performed in DMSO/HCl (3 mM) (1 : 9, pH 2.5) at 130°C for 20 min, successfully leading to the oxime-coupled** 25** in RCY of 56–93%. Noteworthy, the Boc-protective group was removed thermolytically during the radiolabeling reaction. Finally, the ^18^F-labeled RGD glycopeptide** 25** was studied in M21 tumor-bearing nude mice at 120 min p.i. Compared to** 24** [32], this [^18^F]FDG-RGD (**25**) also showed a relatively high accumulation in the heart (0.9% ID/g) and a high uptake in the tumor (2.2% ID/g) with an excellent tumor-to-blood-ratio of 18.

Al Jammaz et al. used [^18^F]FDG as a building block for the radiosynthesis of folate and methotrexate carbohydrazide conjugates** 26** and** 27** [34]. The respective aminooxy-functionalized precursors (9 mM) were used in DMSO/1% acetic acid/methanol (1 : 1, v/v, pH ∼4.5) at 60°C for 10–15 min. After workup using solid phase extraction (SPE) the product conjugates** 26** and** 27** were obtained in overall RCY of greater than 80% (based on starting [^18^F]FDG), with total syntheses times of approximately 20 min and in specific activities of greater than 9 GBq/*μ*mol. The ^18^F-fluoroglycosylation resulted in folate (**26**) and methotrexate carbohydrazide (**27**) conjugates with log⁡⁡*P* values of −1.5 and −1.6, respectively. Both glycoconjugates were stable in human serum* in vitro* at 37°C for at least 4 hours. Binding affinities to folate receptor-positive KB cells revealed binding characteristics which were superior to binding affinities obtained for the same compounds labeled with radiofluorinated benzene and pyridine prosthetic groups and are comparable to that of native folic acid (*K*_*d*_  (26) = 1.8 nM and *K*_*d*_  (27) = 4.7 nM). In* in vivo* studies using KB tumor-bearing nude mice** 26** showed a favorable biodistribution profile with low uptake in intestine, liver, and kidney, rapid clearance from the blood, and high specific uptake in the tumor, resulting in tumor-to-blood and tumor-to-muscle ratios of 11.07 and 9.22, respectively.

[^18^F]FDG has also been used for the ^18^F-fluoroglycosylation of multimeric peptides applying the oxime formation strategy [35]. In this study, monomeric, dimeric, and tetrameric neurotensin (8-13) was aminooxy-functionalized and coupled to [^18^F]FDG in methanol/water (2 : 1, v/v). After 30 min at 80°C the RCY was 63% or 80% when using 3 mM or 7.5 mM peptide, respectively. A decreased RCY of 15% and 88% was achieved when using 1.4 mM or 4 mM of the dimeric neurotensin (8-13) derivative. With the tetrameric derivative only low RCY of 5–8% were achieved, probably due to the very low concentration of the aminooxy-functionalized peptide (0.7–2 mM). The monomeric labeling product could be separated from [^18^F]FDG by radio-HPLC; however, the authors did not give any information on the specific activity of the final ^18^F-labeled glycopeptide.

In the endeavor to improve the oxime conjugation step, 5-[^18^F]fluoro-5-deoxyribose ([^18^F]FDR) has been considered as an alternative prosthetic group (Scheme 4) [36, 37, 55, 56]. The idea is that, in comparison to [^18^F]FDG, the 5-membered ring sugar [^18^F]FDR with the fluorine at C-5 instead of C-6 favors the ring opening of the sugar to the aldehydic form and therefore promotes the oxime ligation with aminooxy-functionalized peptides even under mild reaction conditions, such as ambient temperature and less acidic pH of 4.6.

[^18^F]FDR has been synthesized starting from methyl 2,3-*O*-isopropylidene-5-*O*-(*p*-toluenesulfonyl)-*β*-D-ribofuranoside (**36**, Scheme 4) using standard kryptate-based ^18^F-labeling conditions. After HPLC isolation, which turned out to be essential for separation of free ribose from the ^18^F-labeled product, the resulting methyl 2,3-*O*-isopropylidene-5-deoxy-5-[^18^F]fluororibofuranoside (**37**, Scheme 4) is hydrolyzed with aqueous HCl and purified by solid phase extraction. The average RCY for [^18^F]FDR is about 35% and the radiosynthesis takes 85 min [37, 55]. This ribose-free sugar was used for the conjugation of the two aminooxy-functionalized RGD peptides c(RGDfK) and c(RGDfC) [36], which were conjugated at room temperature in sodium acetate buffer at pH 4.6. With a peptide concentration of 10 mM the achieved RCY were 65–92% after 15 min. The radiolabeled products** 29** and** 30** were purified by radio-HPLC; however, the specific activity of the final ^18^F-glycopeptide was not reported. Cell binding experiments were performed, revealing specific binding of** 29** and** 30** to *α*_*v*_ *β*_3_-expressing PC3 cells.

Moreover, the ^18^F-fluoroglycosylation by the use of [^18^F]FDR was also demonstrated using sialic acid-binding Ig-like lectin 9 (siglec-9) [37]. Siglec-9 is peptide targeting vascular adhesion protein 1 (VAP-1) which is a unique target in inflammatory processes. Performing the oxime formation reaction with [^18^F]FDR in sodium acetate buffer (pH 4.6, 90 mM) at room temperature, the peptide concentration required for an adequate RCY was 15 mM, which was not applicable in the case of a 2 kDa peptide. Applying an anilinium buffer (pH 4.6) instead, the required peptide concentration could be reduced to 0.3 mM and the conversion at room temperature was 50–60% after 10 min. The ^18^F-labeled glycopeptide [^18^F]FDR-Siglec-9 (**31**) was isolated by HPLC and was prepared in a total synthesis time of 120 minutes in an overall RCY of about 27% (referred to [^18^F]fluoride at EOB) with specific activities of 36–43 GBq/*μ*mol. Finally, Li et al. showed that** 31** was suitable for the visualization of the inflammation focus in rats with turpentine oil induced inflammation.

Another approach using [^18^F]FDG and [^18^F]FDR for oxime formation with target molecules aimed at the synthesis of cannabinoid ligands for PET imaging of the cannabinoid receptors 1 (CB1) and 2 (CB2) [38]. Therefore, hydroxylamine-functionalized Rimonabant-type pyrazoles were conjugated to [^19^F]FDG and [^19^F]FDR and affinities for the CB1 and CB2 receptors were determined. In this study, FDR proved to be superior to FDG conjugation, as the conjugation occurred under milder conditions and at higher reaction rate (room temperature, 20 min versus 100°C, 30 min). Compared to NESS125A, which was used as lead structure, the resulting fluoroglycosylated ligands** 32**–**35** showed only weak affinities to CB1 (540–720 nM) and CB2 (310–1400 nM) and low subtype-selectivity (CB2 : CB1 = 0.7–2). Thus, the ^18^F-radiosyntheses of these compounds were not performed by the authors.

## 4. Miscellaneous **^**18**^**F-Fluoroglycosylation Reactions

The first study dealing with the idea of using [^18^F]FDG as ^18^F-fluoroglycosylating agent was reported by Prante et al. in 1999 [39] aiming at a highly selective and mild ^18^F-labeling method of biomolecules by enzymatic ^18^F-fluoroglycosylation. The authors succeeded in the radiosynthesis of UDP-2-deoxy-2-[^18^F]fluoro-*α*-D-glucopyranose (UDP-[^18^F]FDG,** 38**) as a substrate for glycosyltransferases. The radiosynthesis started from 1,3,4,6-tetra-*O*-acetyl-2-deoxy-2-[^18^F]fluoroglucopyranose as an easily available intermediate in the [^18^F]FDG synthesis, which was converted to [^18^F]FDG-1-phosphate by MacDonald phosphorylation and further allowed to react by enzymatic activation to obtain UDP-[^18^F]FDG (**38**) in a RCY of 20% (based on [^18^F]fluoride) after a total synthesis time of 110 min [40]. UDP-[^18^F]FDG (**38**) was obtained in aqueous medium in the void volume of a solid phase cartridge for further glycosyltransferase-mediated reactions.

Bormans and Verbruggen used an enzymatic approach with an* in situ* glycosyl transfer reaction for the synthesis of 2′-[^18^F]fluorodeoxylactose (**39**) [41]. They applied an enzymatic method starting from [^18^F]FDG in a mixture of MnSO_4_, *α*-lactalbumin, galactosyltransferase, and UDP-galactose in HEPES buffer (pH 7.5) at 37°C for 3 h, providing 2′-[^18^F]fluorodeoxylactose after HPLC isolation in a RCY of 3.4%. To evaluate its usefulness for* in vivo* visualization of LacZ gene expression biodistribution studies were performed in normal mice and in Rosa-26 mice that express bacterial LacZ in most of their tissues.** 39** was cleared by urinary excretion and was not retained in any particular organ, neither in normal nor in Rosa-26 mice, suggesting that** 39** is not able to cross the cell membrane.

Phenix et al. used an analogue of [^18^F]FDG in an enzymatic approach for tagging acid *β*-glucocerebrosidase (GCase), a recombinant enzyme formulated in Cerezyme which is used to treat Gaucher disease [42]. In this innovative method 2,4-dinitrophenyl-2-deoxy-[^18^F]-2-fluoro-*β*-D-glucopyranoside (*β*-DNP-[^18^F]FDG) is formed from [^18^F]FDG and 1-fluoro-2,4-dinitrobenzene in aqueous NaHCO_3_/ethanol (1 : 1, v/v) in a one-step reaction at 37°C yielding the ^18^F-labeled product in 85% RCY after 10 min. *β*-DNP-[^18^F]FDG was isolated by HPLC and the enzymatic approach was evaluated with the test-enzyme *β*-glucosidase from* Agrobacterium *sp. as well as with the therapeutically relevant enzyme GCase. ^18^F-labeling of glucosidase from* Agrobacterium *sp. proceeded within a few minutes in high RCY, whereas ^18^F-labeling of GCase was hampered by the much higher *K*_*i*_ value. ^18^F-labeled GCase (**40**) was obtained in a total synthesis time of about 2.5 h in a specific activity of ∼2 GBq/*μ*mol and was used to monitor the biodistribution of GCase in mice. The highest uptake was observed in macrophage-rich organs, such as the liver and spleen, as well as in the gall bladder, kidneys, intestines, heart, and femur, indicating elimination of** 40** through renal and hepatobiliary routes. Almost no radioactivity was detected in the brain indicating high stability of ^18^F-Glc-GCase (**40**) as enzymatic turnover or proteolytic degradation would result in free [^18^F]FDG which would show high uptake in the brain. The biodistribution and PET imaging studies on animals revealed that ^18^F-labeled GCase (**40**) is a powerful tool for monitoring the enzyme distribution and tissue half-life* in vivo* by PET with an immediate clinical application to Gaucher disease. The authors conclude that the ^18^F-labeling method, starting from [^18^F]FDG, could be adapted to alternative enzymes, opening the path for application to a variety of enzyme replacement therapies.

In one of the earlier studies on ^18^F-fluoroglycosylation reactions, the radiosynthesis of a new carbohydrate-conjugated 2-nitroimidazole derivative starting from peracetylated [^18^F]FDG as potential agent for tracking hypoxic tissues was reported by Patt et al. [43]. Peracetylated [^18^F]FDG was isolated by semipreparative HPLC and then allowed to react with 2-nitroimidazole (88 mM) in the presence of Hg(CN)_2_ and SnCl_4_ in acetonitrile at 70°C for 60 min to give the ^18^F-labeled product in high RCY of 80%. After a second HPLC isolation and Zemplén deacetylation [57] with sodium methylate in methanol the glucose derivative** 41** was subjected to cell uptake experiments* in vitro* and biodistribution studies in tumor-bearing rats. However, the uptake of the ^18^F-glucosylated nitroimidazole** 41** in cells under normoxic conditions was very low (0.1-0.2%) and the* in vivo* data did not indicate significant tumor uptake, rendering this radiotracer unsuitable for the detection of hypoxic tissues* in vivo* by PET.

A thiol-reactive ^18^F-glucosyl derivative for the site-specific ^18^F-fluoroglycosylation of peptides was developed by Prante et al. [44], applying the chemoselective thiol substitution reaction of mixed thiols. Aiming at the synthesis of ^18^F-labeled glycosyl thiosulfonates as a “mixed thiol” analog, peracetylated [^18^F]FDG was isolated by HPLC and converted to the corresponding bromide [58, 59] and subsequently allowed to react to the 1-phenylthiosulfonate using sodium phenylthiosulfonate (NaPTS) and tetrabutylammonium bromide in acetonitrile/DMF (4 : 1, v/v). Ac_3_[^18^F]FGlc-PTS was obtained in a RCY of 33% in a synthesis time of 90 min (related to [^18^F]fluoride) which was further used for the chemoselective ^18^F-fluoroglycosylation of thiols, that is, the model peptide CAKAY and the thiol-bearing cyclo-RGD peptide c(RGDfC). The ^18^F-fluoroglycosylation proceeded chemoselectively with 1 mM peptide in tris-buffer (pH 7.7)/acetonitrile (4 : 1, v/v) at room temperature and in high RCY of >90% after 15 min. The total radiosynthesis, including the preparation of the ^18^F-fluoroglycosylating reagent Ac_3_-[^18^F]FGlc-PTS, peptide ligation, and final HPLC purification, provided a non-decay-corrected yield of 13% after 130 min. The stability of the ^18^F-fluoroglycosylated RGD peptide** 42** was verified in human serum* in vitro* showing no cleavage of the carbohydrate moiety for at least 90 min.

Transferring this method to the ^18^F-fluoroglycosylation of proteins, an approach for site-specific conjugation of thiol-functionalized [^18^F]fluorosugars to cysteine (Cys) or dehydroalanine (Dha, directly accessible from Cys) tagged proteins was reported by the Davis group [45]. Direct thionation of [^18^F]FDG was achieved using Lawesson's reagent in 1,4-dioxane at 100°C during 45 min, resulting in the formation of 2-deoxy-2-[^18^F]fluoro-1-thio-glucopyranose in 98% RCY. In a one-pot procedure 2-deoxy-2-[^18^F]fluoro-1-thio-glucopyranose was directly used for mixed disulfide formation with a Cys-bearing protein (3 *μ*M) or conjugate addition to a Dha-bearing protein (0.85 *μ*M) in sodium phosphate buffer (pH 8.0) at room temperature or 37°C. The RCY for the site-specific labeling procedure were between 40 and 60% after 15 min. Starting from [^18^F]FDG, SS- and S-linked 2-[^18^F]fluoroglycoproteins** 43** and** 44** were synthesized in overall RCY of 55–60% after a total synthesis time of 90 min. The suitability of such ^18^F-fluoroglycosylated proteins for the application as PET imaging agents still has to be shown.

In an attempt to synthesize a PET-MR hybrid imaging agent [^18^F]FDG was conjugated to magnetic iron oxide nanoparticles (MNPs) [46]. The labeling precursor 3,4,6-tri-O-acetyl-2-*O*-trifluoromethanesulfonyl-(*N*-(2-mercaptoethyl))mannopyranosylamine was synthesized by reductive amination from the precursor 1,3,4,6-tetra-*O*-acetyl-2-*O*-trifluoromethanesulfonyl-*β*-D-mannopyranose (mannose triflate) with 2-aminoethanethiol. The ^18^F-fluorination was performed using standard labeling conditions in DMF at 90°C for 20 min. SPE purified 2-deoxy-2-[^18^F]fluoro-(*N*-(2-mercaptoethyl))-*β*-D-glucopyranosylamine was added to a FeCl_3_ solution (25 mM), followed by NaBH_4_ (1 M) at 60°C until pH 9 was reached. Black MNPs were formed in 2 h, which were separated from the mixture by a magnet. A similar attempt was performed by the same group for the radiosynthesis of [^18^F]FDG conjugated gold nanoparticles** 45** that were further conjugated to an anti-metadherin antibody for targeting breast cancer cells [47].

## 5. Conclusion

There are several methodologies to introduce an ^18^F-fluoroglycosyl residue into a biomolecule. Besides enzymatic or thiol-selective reactions the most frequently used ones are the ^18^F-fluoroglycosylation via CuAAC and via oxime formation. One drawback of the CuAAC supported glycosylation is the fact that the labeling precursor 3,4,6-tri-*O*-acetyl-2-*O*-trifluoromethanesulfonyl-*β*-D-mannopyranosyl azide (**1**) is not commercially available and that its synthesis is challenging. This was circumvented by the development of the new and very easy to synthesize precursor 2,3,4-tri-*O*-acetyl-6-*O*-tosyl-glucopyranosyl azide** 19** which has the additional advantage that, at least in the case of glycosylated RGD peptides [30], the resulting 6-deoxy-6-[^18^F]fluoroglucosyl conjugates have favorable bioproperties compared to the 2-deoxy-2-[^18^F]fluoroglucosyl derivatives.

The major advantage of the ^18^F-fluoroglycosylation via oxime formation is the fact that widely available [^18^F]FDG can be used; the limitations are the difficulties associated with the synthesis and stability of biomolecules containing aminooxy moieties and the harsh reaction conditions (high temperature, low pH) that are required for sufficient RCY. The use of [^18^F]FDR needs much milder reaction conditions, but then the advantage of easy accessible [^18^F]FDG is forfeited.

In conclusion, several examples published in the last few years have shown that ^18^F-fluoroglycosylation is a powerful and highly valuable tool for the radiosynthesis of ^18^F-glycoconjugates with suitable* in vivo* properties for PET imaging studies.

## Figures and Tables

**Table 1:** ^
18^F-Fluoroglycosylated imaging probes and ^19^F-fluoroglycosyl derivatives (*) synthesized via CuAAC.

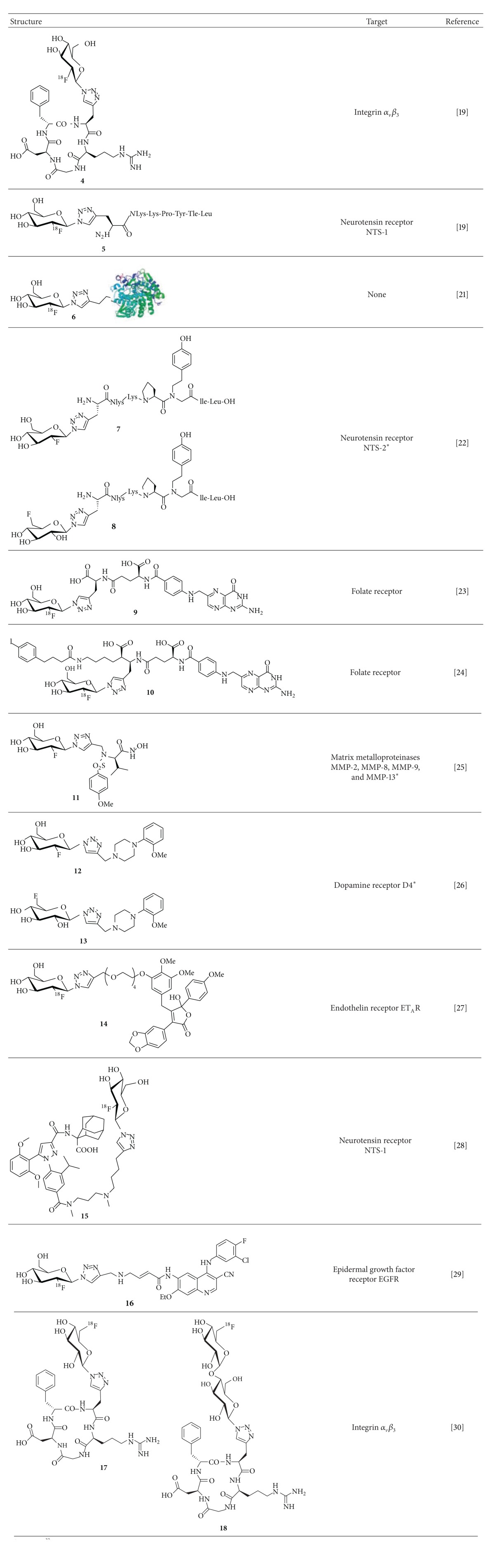

*Only the ^19^F-compounds were synthesized.

**Table 2:** ^
18^F-Fluoroglycosylated imaging probes and ^19^F-fluoroglycosyl derivatives (*) synthesized via oxime formation.

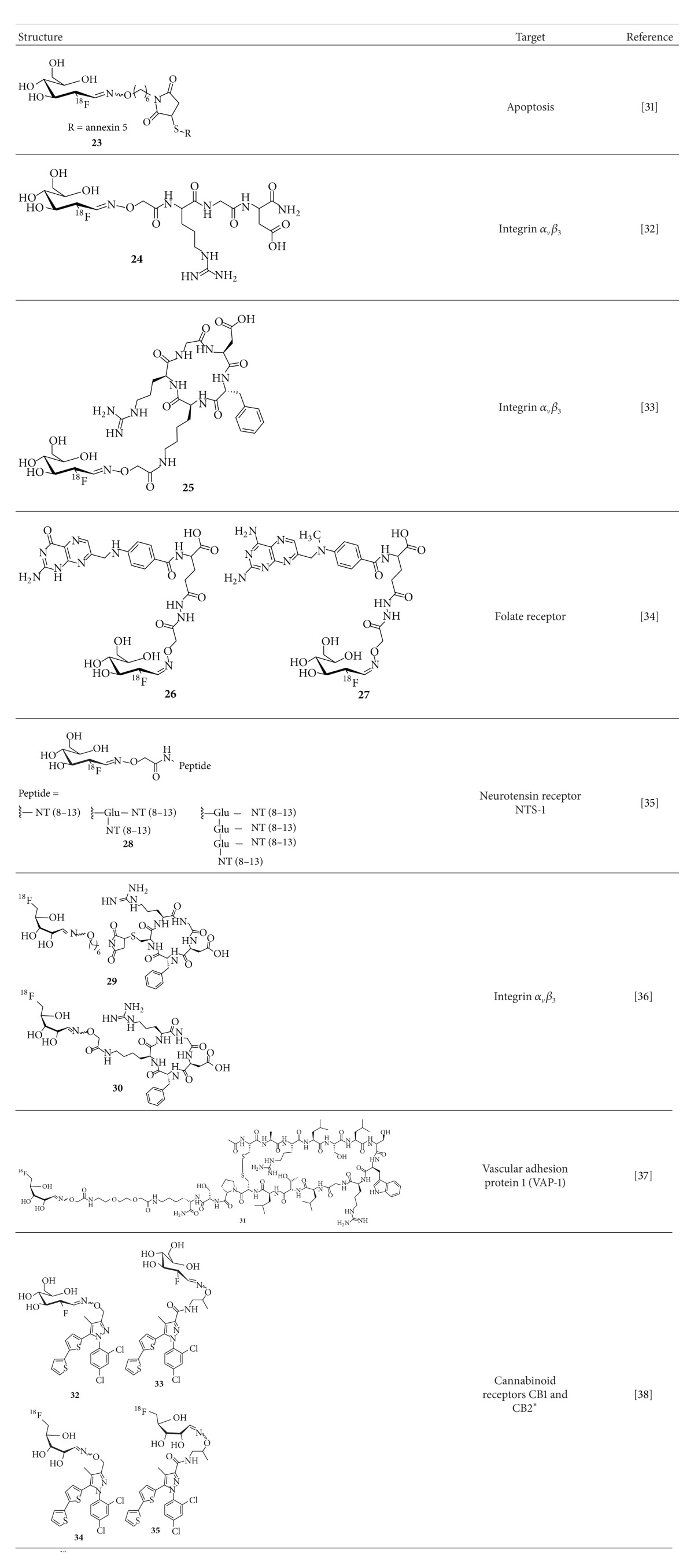

*Only the ^19^F compounds were synthesized.

**Table 3:** ^
18^F-Glycoconjugates synthesized by miscellaneous ^18^F-fluoroglycosylation.